# Lignin Inter-Diffusion Underlying Improved Mechanical Performance of Hot-Pressed Paper Webs

**DOI:** 10.3390/polym13152485

**Published:** 2021-07-28

**Authors:** Amanda Mattsson, Tove Joelsson, Arttu Miettinen, Jukka A. Ketoja, Gunilla Pettersson, Per Engstrand

**Affiliations:** 1 Department of Chemical Engineering, Mid Sweden University, SE-85170 Sundsvall, Sweden; tove.joelsson@more.se (T.J.); jukka.ketoja@vtt.fi (J.A.K.); gunilla.pettersson@miun.se (G.P.); per.engstrand@miun.se (P.E.); 2 MoRe Research Örnsköldsvik AB, Box 70, SE-89122 Örnsköldsvik, Sweden; 3 Department of Physics, University of Jyvaskyla, P.O. Box 35, FI-40014 Jyvaskyla, Finland; arttu.i.miettinen@jyu.fi; 4 VTT Technical Research Centre of Finland Ltd., P.O. Box 1000, FI-02044 Espoo, Finland

**Keywords:** hot-pressing, paper web, fibre, lignin, diffusion, activation energy

## Abstract

Broader use of bio-based fibres in packaging becomes possible when the mechanical properties of fibre materials exceed those of conventional paperboard. Hot-pressing provides an efficient method to improve both the wet and dry strength of lignin-containing paper webs. Here we study varied pressing conditions for webs formed with thermomechanical pulp (TMP). The results are compared against similar data for a wide range of other fibre types. In addition to standard strength and structural measurements, we characterise the induced structural changes with X-ray microtomography and scanning electron microscopy. The wet strength generally increases monotonously up to a very high pressing temperature of 270 °C. The stronger bonding of wet fibres can be explained by the inter-diffusion of lignin macromolecules with an activation energy around 26 kJ mol^−1^ after lignin softening. The associated exponential acceleration of diffusion with temperature dominates over other factors such as process dynamics or final material density in setting wet strength. The optimum pressing temperature for dry strength is generally lower, around 200 °C, beyond which hemicellulose degradation begins. By varying the solids content prior to hot-pressing for the TMP sheets, the highest wet strength is achieved for the completely dry web, while no strong correlation was observed for the dry strength.

## 1. Introduction

Microplastic emissions are one of the world’s greatest environmental threats. The amount of these emissions has been steadily increasing for many years and is expected to continue to do so [1]. Thus, material options that are both renewable and biodegradable have been extensively searched for. A particular challenge is to develop materials that have similar or better properties in humid or wet conditions as their oil-based counterparts. This should be the case not only for strength but also for dimensional stability and barrier properties, which are important, e.g., in packaging and construction applications [2].

Recent studies have shown that hot-pressing of lignin-rich paper webs could provide at least a partial solution to the above challenge. Clear improvements are observed for both wet and dry tensile strength (later also referred to as only wet and dry strength) compared to non-treated webs [3,4], enabling applications in several packaging areas. Joelsson et al. [5] showed that the tensile strength of paper based on chemithermomechanical pulp (CTMP) could be improved even by 100% when passing the paper through a hot nip (200 °C, 6 MPa) with a pressing time of 1.5 s and 70 s after hold. Moreover, by hot-pressing, the wet strength increased dramatically to a value of about 16 kNm/kg from the level of 2 kNm/kg found for non-heat treated paper. It could be seen that the amount of lignin was of great importance [5,6]. Thus, such a hot-pressing technology could provide innovative product solutions once both suitable raw materials and optimal process conditions are defined by a deeper understanding of underlying strengthening mechanisms. Also, other properties that are important for the final applications of packaging papers, such as water resistance, have shown promising results. Contact angle measurements showed increased values for the hot-pressed paper samples [5], which suggests a more hydrophobic surface [7]. Similar results and conclusions, that heat treatment of this material increases the hydrophobicity, have been observed in the area of thermal modification and welding of wood [8,9].

Joelsson et al. [5] postulate that softened lignin from fibres redistributes within the consolidated structure, enabling strong inter-fibre bonding even in a wet fibre network. In other words, lignin acts as a natural wet-strength additive. Similar heat-induced bonding was already found by Gupta et al. in 1962 [10]. They applied isolated lignin to paper samples and pressed them together at high temperature. The appearing inter-layer bonding increased the strength properties. The optimal bonding temperature depended on lignin type and differed for wet or dry paper. This was explained by the thermal transition of lignin and by the plasticizing effect of water, which reduces the glass transition temperature (Tg) by 70–165 °C depending on the type of lignin [11].

The importance of water for the viscoelastic properties of wood was reviewed in 1982 by Back and Salmén [12], who concluded that water-saturated native lignin has a softening temperature of about 115 °C. This could be further lowered by sulphonation. Joelsson et al. [13] have recently shown that the softening effect of sulfonation also occurs when a sulfite-enriched paper is hot-pressed. In this case, a lower temperature is required to maintain strength.

Lignin is often referred to as a by-product in industries such as the production of paper, ethanol, biomass, etc. [14]. However, the polymer is seen to have a huge technological potential, and related research has expanded in recent years. Nevertheless, there are currently only a few commercial products based on lignin. For example, there are pulping processes where the lignin is not totally removed, leading to so-called high yield pulps (HYP). Their yield can be as high as 95%, which can be compared to the yield of about 50% for chemical pulp with removed lignin [15]. Thus, high yield pulping is a preferred option from the viewpoint of efficient utilisation of wood raw material. The main reason for removing the lignin in chemical pulping is to achieve high brightness and strength, which are important properties, e.g., white packaging, copy paper and some heavily-coated brochure papers. On the other hand, HYP and particularly thermomechanical pulp (TMP) are mainly used for magazine paper, newspaper and book paper, for which high opacity and light scattering are more important than brightness or strength. However, the share of paper usage has declined rapidly during recent years, which leaves a considerable amount of space for new markets. At the same time, the process targets should be reconsidered based on the changed product requirements.

The aim of this work is to investigate mechanisms underlying the above improved mechanical properties obtained by hot-pressing. In particular, we would like to know how to control lignin redistribution in fibre networks without deteriorating other fibre polymers such as hemicelluloses. Moreover, the high temperature and moisture content of fibres may introduce also other structural changes that affect the mechanical properties of the hot-pressed web. These changes are characterized by X-ray tomography and scanning electron microscopy (SEM). The experimental results are interpreted with the help of theoretical ideas on polymer inter-diffusion. In addition to analysing the results carefully for webs containing TMP, we show that the same diffusion mechanisms explain the wet-strength improvement for a wide variety of other pulps despite their different lignin content.

## 2. Materials and Methods

### 2.1. Materials

The paper materials used in this study were based on different mechanical and chemical kraft pulps obtained from Swedish mills, together with some pulps produced in a laboratory. Mechanical pulps with a lignin content of 26–28% included the following types: TMP (Holmen AB Braviken mill, Norrköping, Sweden), CTMP (Rottneros AB Rottneros mill, Sunne, Sweden, and SCA AB Östrand mill, Timrå, Sweden), and high-temperature chemithermomechanical pulp (HTCTMP) produced at the test pilot refinery at Valmet AB, Sundsvall, Sweden. Chemical kraft pulps with a lignin content of 0–12% were unbleached kraft liner (SCA AB Obbola mill, Umeå, Sweden), bleached kraft liner (Metsäboard Husum mill, Örnsköldsvik, Sweden), bleached kraft (Södra Cell Värö mill, Varberg, Sweden), and unbleached kraft with different rest-lignin contents produced at the laboratory pilot of MoRe Research (Örnsköldsvik AB, Örnsköldsvik, Sweden). The mechanical pulps and the pilot-produced chemical kraft pulps were based on Norway spruce, and the rest of the chemical pulps were based on softwood (a mixture of spruce and pine). The lignin content was measured by the Klason method (T222).

All paper material except that containing TMP were prepared using a Rapid Köthen sheet former (Paper Testing Instruments, Pettenbach, Austria) according to ISO 5269-2:2004, resulting in uniform fibre orientation. The TMP paper was produced in an XPM Fourdrinier paper machine at the laboratory of MoRe Research (Örnsköldsvik AB, Örnsköldsvik, Sweden). The web width was 0.225 m, the machine speed was 1.4 m/min, and the fibre orientation ratio was 1.7 between machine direction (MD) and cross-machine direction (CD). The grammage of the paper materials was in the range of 100–150 g/m^2^. In both of the above production methods, the structure forming step is followed by water removal with wet-pressing at relatively low temperatures, which significantly affects the density of the formed paper material. However, the largest changes in density take place during the final hot-pressing process.

### 2.2. Pressing Methods

**Figure 1:**
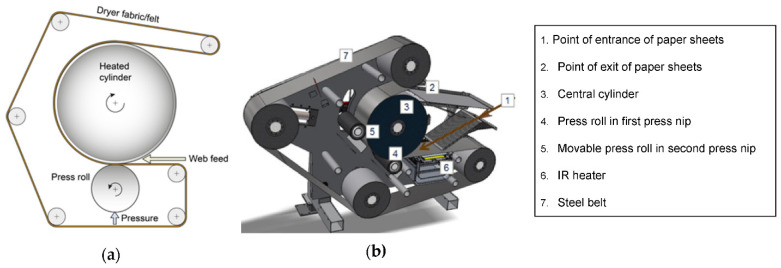
(**a**) Felted, oil-heated cylinder press [16] operated at MoRe Research Örnsköldsvik AB, Örnsköldsvik, Sweden. Illustration: Mats Rundlöf, Capisco, Norrköping, Sweden. (**b**) Infrared-heated press based on a steel belt [17] produced by Ipco AB, Sandviken, Sweden. Both pictures are reproduced under the terms of the CC BY license.

Two different pressing methods were applied in the experiments (Figure 1). Firstly, test points pressed at temperatures equal to or lower than 200 °C were performed using an oil-heated cylinder press (Figure 1a). Moist sheets were fed into the press on a felted fabric with a rate of 1 m/min and a nip pressure of 6 MPa. The pressing time in the nip was 1500 ms (at a nip length of about 25 mm) and after-hold was 70 s. Secondly, test points hot-pressed at temperatures higher than 200 °C were performed using a test pilot press with an infrared-heated steel belt carrying the paper samples through a nip shown in Figure 1b. The speed was 3 m/min, corresponding to a pressing time of 40 ms (nip length was about 2 mm) and the after-hold was 23.5 s. The nip load was estimated to be 8 MPa, and the press load of the steel belt was 0.15 MPa. In both cases, nip lengths were measured with sensor films from Fujifilm Holdings Corporation (Tokyo, Japan), Prescale LW 2.5–10 MPa. Thin blotter papers on both sides of an actual paper sample were used in all tests to prevent sticking. The solids content of paper sheets was 50–60% before pressing at the cylinder press, and at the infrared-heated steel belt press test pilot solids content of TMP sheets was 50–100%.

### 2.3. Sheet Testing

Sheet testing was carried out after conditioning according to ISO 187. Grammage and density were determined according to ISO 536 and ISO 534 respectively. The standard sheet thickness was measured according to ISO 5270. Dry tensile strength was determined according to ISO 1924-3. Wet tensile strength was measured according to ISO 3781 after immersion in water for one hour.

### 2.4. Characterisation

#### 2.4.1. Scanning Electron Microscope (SEM)

Image analyses using a high-resolution SEM (Tescan Maya3-2016, TESCAN Brno, s.r.o., Brno, Czechia) were performed on TMP sheets with different pressing conditions. The applied electron beam voltage was 3.00 kV and the beam intensity was 1.00. To obtain images of the structures at different scales, magnifications 500×, 2000× and 10,000× were used. These magnifications correspond to pixel sizes of 270 nm, 67 nm and 13 nm, respectively. The corresponding beam spot sizes were 26 nm, 26 nm and 4 nm, respectively. A secondary electron detector was used to capture the images. The working distance to the sample, which ranges from 5 to 7 mm, was adjusted for each image to achieve the best possible image quality.

The cross-sections were polished either using an argon ion milling system (Hitachi IM4000Plus, Hitachi High-Tech Co., Tokyo, Japan) or by freeze-drying the specimens at −110 °C and vacuum for 12 h followed by crushing to produce the transverse sections. Lastly, the samples were prepared by sputtering them with a 5–10 nm thin layer of iridium prior to imaging.

#### 2.4.2. X-ray Microtomography

X-ray tomography images of the sheets pressed at different temperature levels were acquired using an X-ray microtomograph (CT) (Xradia MicroXCT-400, Xradia Inc., Concord, CA, USA). A sample approximately 1 mm wide was cut from the sheet with a surgical knife and glued to the top of a carbon fibre rod, which served as a sample holder. Images were acquired with 0.6 µm pixel size, corresponding to 1.5 µm spatial resolution (MTF10%), at 40 kV X-ray tube accelerating voltage and 4 W power. 1750 projection images per sample were acquired with an exposure time of 10 s per projection. The projections were reconstructed into a 3D volume image using the filtered backprojection algorithm. The volume images show an area of approximately 1.1 mm × 1.1 mm of the sheet.

**Figure 2:**
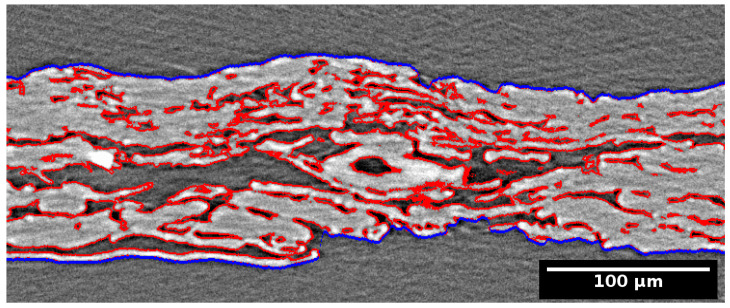
Visualisation of the X-ray microtomograph (CT) image of a sample pressed at 190 °C temperature (grayscale), edges of segmented regions (red) and surfaces of the sheet (blue).

The reconstructed images were denoised using bilateral filter (spatial sigma = 1.5 µm, radiometric sigma ≈ 7% of dynamic range) [18]. The filtered images showed a high contrast-to-noise ratio (typically ≈ 40) and could therefore be segmented using the simple Otsu thresholding method [19]. After the thresholding procedure, the remaining small image artefacts were removed by deleting all contiguous regions whose size was less than 100 voxels. This procedure results in a visually correct segmentation, as shown in Figure 2.

The surfaces of the paper sheets were defined using the Carpet method [20] which works by lowering a surface following quenched noise Edwards-Wilkinson dynamics towards the segmented paper sheet. The bright pixels corresponding to the paper eventually slow down and stop the evolution of the surface. The paper surface is then defined by the position where the motion of the dynamic surface stops. An example of the surfaces is shown in Figure 2.

The total volumes of the sheet, pores, and fibres were determined by counting the number of pixels classified to each material phase. The pore size distribution was determined using the local thickness algorithm [21]. Image analysis was performed using the freely available software pi2 (https://www.github.com/arttumiettinen/pi2, accessed on 28 July 2021), and 3D visualisations were created using MeVisLab (MeVis Medical Solutions AG, Bremen, Germany).

## 3. Results

### 3.1. Porosity of the Fibre Networks from X-ray Microtomography

**Figure 3:**
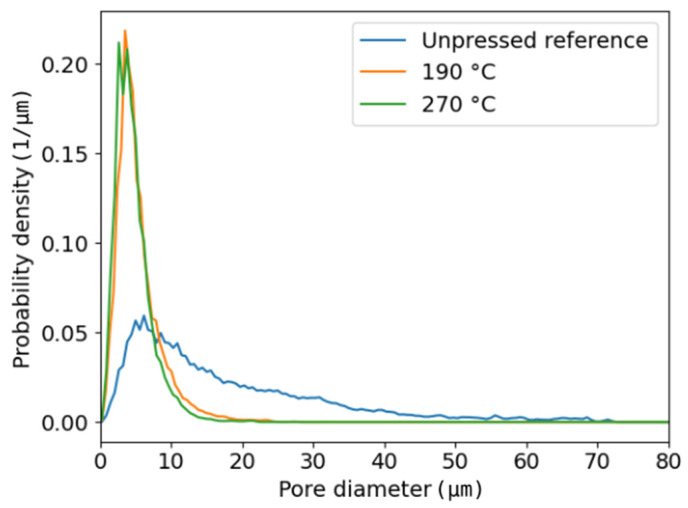
Pore-size distributions for the unpressed reference sheet and hot-pressed sheets with temperature of 190 °C (cylinder press) and 270 °C (steel belt press).

Hot-pressing narrows the pore-size distribution of a sheet significantly as can be seen in Figure 3. This effect is strongest at very high temperatures. Still, the mean pore size in all cases is several micrometres and thus clearly higher than the resolution of X-ray imaging. Therefore, it is reasonable to assume that the measurement of total pore volume gives reliable results.

**Figure 4:**
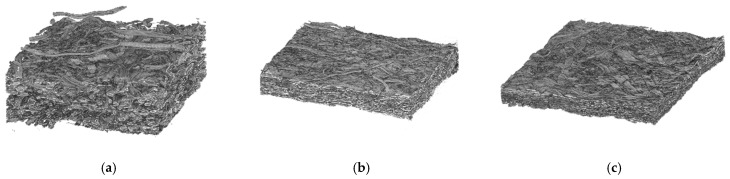
3D visualisations of the CT images of samples. (**a**) Unpressed reference, (**b**) pressed at 190 °C, and (**c**) pressed at 270 °C.

The average porosity, 0.74, is quite high for the unpressed reference sheet. In this case, lumens are still partly open, and the above value for porosity is similar as in earlier similar measurements [22]. During hot-pressing, density increases and porosity decreases significantly as lumina collapse and fibres soften (Figure 4). Moreover, a slight decrease in sheet porosity is also observed when temperature and pressure are increased, from 0.34 at 190 °C and 6 MPa (cylinder press) to 0.32 at 270 °C and 8 MPa (steel belt press), despite the much shorter pressing time in the latter case. Thus, plastic fibre deformations take place very rapidly at high temperatures when the polymer components of fibres soften.

Table 1 shows the resulting sheet densities. The values obtained from 3D structural images are higher than those obtained with standard sheet density measurements mainly because of the surface roughness volume excluded when calculating the effective value from the CT data.

The effective fibre density $\rho_{f}$ can be measured from sheet grammage G, area of sample A, and total volume of fibres $V_{f}$ determined from the 3D images,

(1) $$\rho_{f}=\frac{GA}{V_{f}}$$

Equation (1) gives the values 1440 kg/m^3^ (unpressed reference), 1450 kg/m^3^ (190 °C, cylinder press) and 1460 kg/m^3^ (270 °C, steel belt press) for the density of the fibres. The wall density without lumen is about 1500 kg/m^3^ for natural wood fibres [23]. The slightly lower values can be explained by a small total volume of pores whose size is below the imaging resolution. However, the main conclusion is that the hot-pressing does not induce any noticeable density change in the fibre walls, despite a large reduction in the network porosity and mean pore size.

### 3.2. Visual Observations on Pressing-Induced Changes in Fibres

**Figure 5:**
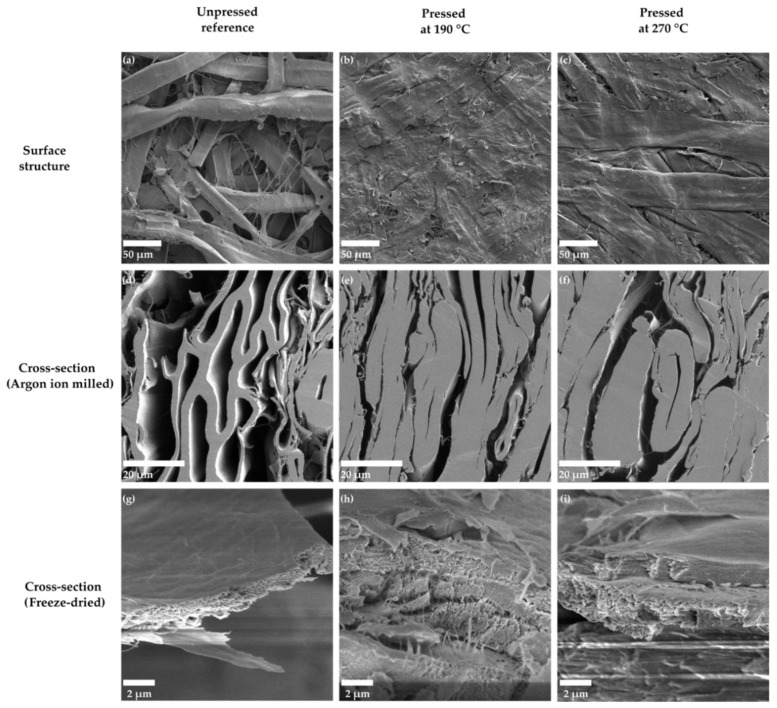
SEM images of the structures for three different pressing temperatures for the material, (**a**) unpressed reference, (**b**) pressed at 190 °C, and (**c**) pressed at 270 °C. SEM images of the cross-sections polished with an argon ion miller for three different pressing temperatures for the material, (**d**) unpressed reference, (**e**) pressed at 190 °C, and (**f**) pressed at 270 °C. SEM images of the freeze-dried cross-sections of the fibre wall for three different pressing temperatures for the material, (**g**) unpressed reference, (**h**) pressed at 190 °C, and (**i**) pressed at 270 °C. The working distance for the samples was in the range from 5 to 7 mm.

Figure 5 shows SEM images of the TMP paper sheets pressed at different temperatures. The unpressed sample (Figure 5a) has a porous structure, with fibres having their characteristic oval shape. For the sheets pressed at higher temperatures, 190 °C (Figure 5b), and 270 °C (Figure 5c), fibres consolidate into ribbon-like structures with an almost perfectly closed lumen. The sample pressed at 190 °C (cylinder press) is treated for a much longer time, 1.5 s in the nip and 70 s after hold, compared to the sample at 270 °C (steel belt press), treated 40 ms in the nip and 23 sec in after hold. This difference appears as a more closed surface for the 190 °C sample, despite its slightly higher overall porosity (see Section 3.1).

The porosity differences in different samples are best visible in SEM cross-sections of these structures, obtained after polishing the samples with an argon ion milling machine. In addition to inter-fibre pores, also fibre lumens stay partly open for the unpressed sheets (Figure 5d). On the other hand, the highest 270 °C temperature causes an almost complete disappearance of lumen space due to thermal softening (Figure 5f), whereas most of the collapsed lumens are still visible at the lower 190 °C temperature (Figure 5e).

In order to look closer at the nano-/microstructure inside the fibre wall, cross-sections were prepared also by freeze-drying the material prior to breaking the sheets. However, in these cross-sections (Figure 5g–i), it is not possible to observe any substantial differences in the porous structure when comparing the unpressed sample and the ones pressed at high temperatures. This suggests that lignin and other matrix polymers are not extracted from the fibre wall to the same extent as for some chemical treatments of wood fibres [24], where the extra microporosity is clearly visible. This observation is in alignment with the fibre wall densities obtained from the CT analyses (see Section 3.1).

### 3.3. Lignin Inter-Diffusion Affecting Wet Tensile Strength

**Figure 6:**
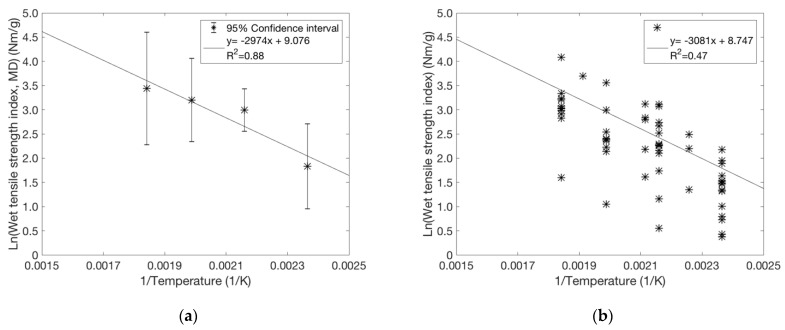
The logarithm of wet tensile strength index plotted against 1/Temperature when pressed with either cylinder press (T = 150 °C, 190 °C; 6 MPa) or steel belt press (T = 230 °C, 270 °C; 8 MPa): (**a**) TMP sheets with preferred MD fibre orientation pressed at an initial solids content of 61%. The points represent an average of 10 data points and their confidence intervals. (**b**) Varied pulps and pressing conditions for standard laboratory sheets with uniform fibre orientation. Solids content varies in the range of 50–65%. The overall trend is described by a similar activation energy of 26 kJ mol^−1^ as in (**a**).

The dependence of wet tensile strength index (wet tensile strength divided by the grammage) on pressing temperature seems to be defined by the activation energy for the inter-diffusion of lignin between fibre surfaces. The inter-diffusion is expected to be proportional to $\exp\left(-\frac{E_{a}}{RT}\right)$ [10], where $E_{a}$ is the activation energy, T is temperature, and R is gas constant. We obtained $E_{a}/R$ by plotting ln(Wet tensile strength index) vs. 1/Temperature (1/T) and taking the slope of the linear fitting line. In Figure 6, this is done first for TMP only (Figure 6a) and then for the whole data (Figure 6b) with different furnishes at temperatures exceeding 150 °C. The relationship between ln(Wet tensile strength index) and 1/T seems quite linear in the range of 150–270 °C for all pulps. This is striking taking into account that the press type and associated nip dwelling time are different below (cylinder press) and above (steel belt press) 200 °C for the data. The above exponential temperature-dependence of lignin diffusion rate thus dominates over other factors when the level of wet tensile strength of pressed material is set by these processes.

The temperature behaviour of TMP (Figure 6a) is quantitatively similar to that of the whole data (Figure 6b) with $E_{a}/R\;$≈ 3080 K, i.e., $E_{a}\;$≈ 26 kJ mol^−1^. This value is close to the value of 29 kJ mol^−1^ obtained earlier for the diffusion of dissolved lignin from the interior of the chip to the bulk liquor, during the kraft pulping of Eucalyptus globulus wood [25]. Thus, the diffusion rate does not seem to be very sensitive to the type of lignin.

**Figure 7:**
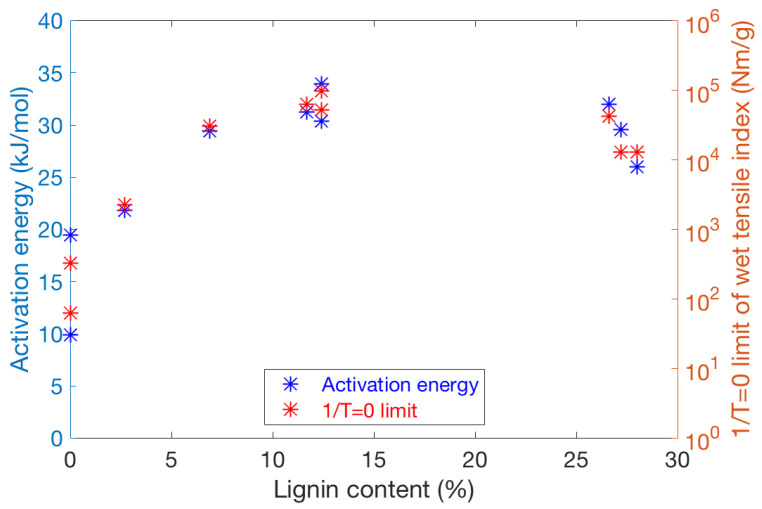
The apparent activation energy (left vertical axis) and extrapolated ln(Wet tensile strength index) at 1/T = 0 (right logarithmic axis) for different pulps with varied lignin content. The extrapolation omits the degradation of fibre-wall polymers and therefore does not describe the true high-temperature limit of wet tensile index. The points up to 12% lignin content describe kraft pulps with varied cooking times in pulping. These results are compared with similar data for CTMP and TMP with lignin content of 26–28%.

We studied the effect of lignin content of fibres by making similar plots for different pulps separately. A systematic increase in lignin content in the range of 0–12% was obtained for chemical kraft pulps by varying the cooking time. The results for these pulps were compared with similar data for CTMP (lignin content 27%) and TMP (lignin content 28%). Figure 7 shows both estimated $E_{a}$ and extrapolated wet strength at 1/T=0 for the different cases. Here the 1/T=0 limit, plotted on a logarithmic axis, describes the order of magnitude of wet strength achievable in hot-pressing. On the other hand, a low $E_{a}$ value seen for the smallest lignin contents indicates a relatively weak temperature dependence, which is generally coupled with a low 1/T=0 limit as well. It seems that at least c.a. 7% of lignin in kraft fibres is required to raise wet strength to a similar level as for the other pulps. On the other hand, lignin content of fibres higher than 12% does not seem to improve wet strength further, as both the activation energy and the 1/T=0 limit saturate in Figure 7. In other words, the main improvement on wet strength is achieved already for moderate lignin content of fibres. This suggests that a fairly thin surface layer of diffused lignin is sufficient to provide the maximal bonding between wet fibres.

**Figure 8:**
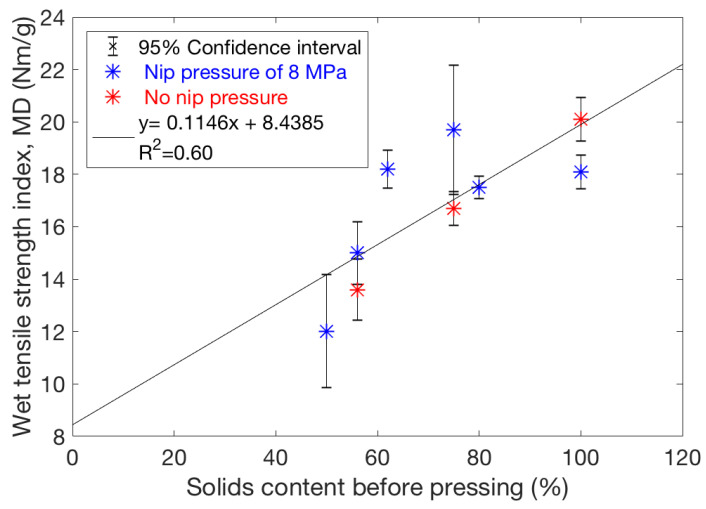
Wet tensile strength index of TMP sheets improves when the pressing is done on a dried web. Here the initial solids content is varied for the constant pressing temperature of 230 °C, keeping other process conditions fixed for all trial points. The points represent an average of 10 data points and their 95% confidence intervals. The applied pressure is 8 MPa for the blue markers, and 0 MPa for the red markers. Wet tensile strength seems not to be very dependent on pressure or density. The applied pressure (0.15 MPa over 23 s) exerted by the steel-belt after the pressing nip seems sufficient to improve wet tensile strength to the same level as when using the 8 MPa nip pressure.

In addition to the primary effects of pressing temperature and lignin content (i.e., pulp type) mentioned above, it is interesting to consider other parameters. Wet strength appears to have a similar level and temperature behaviour for sheets with uniform (Figure 6b) and non-uniform (Figure 6a) fibre orientation. This further suggests that the effective bonding of the contacting inter-fibre surfaces is more important for wet strength than the geometry of the fibre network. This idea is also supported by the observation that wet strength is surprisingly insensitive to nip pressure. When studying heat-treated sheets with and without applied nip pressure, we found no correlation between measured wet strength and average sheet density. On contrary, wet strength and solids content before pressing are correlated as shown in Figure 8. However, the total variation here is much smaller than that for varied temperatures. One possible reason for the correlation between the wet strength index and solids content could be the higher sheet temperature achieved when pressing a drier sheet, which accelerates the lignin inter-diffusion and thus enhances bonding.

### 3.4. Network Stiffness and Dry Tensile Strength

**Figure 9:**
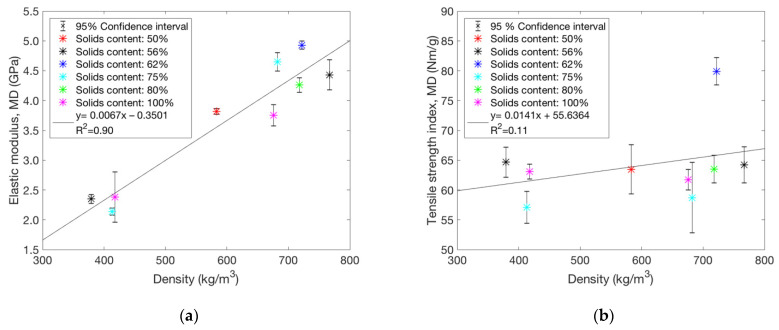
(**a**) Elastic modulus (MD) of hot-pressed sheets with and without nip pressure follows the density as expected. (**b**) Correlation between tensile strength index (MD) and density is still rather weak. The solids contents prior to pressing are indicated in the figures. Note the highest value, which appears like an outlier here, comes from the same measurement as the corresponding point in (**a**). The points represent an average of 10 data points and their 95% confidence intervals.

The same varying solids contents prior to pressing as in Figure 8 were used for the data in Figure 9, where elastic modulus and dry tensile strength index (dry tensile strength divided by the grammage) are compared against sheet density. The elastic modulus for oriented TMP sheets in MD increases with density (Figure 9a). This is expected as density generally determines the relative bond area for random fibre networks [26]. Nevertheless, the correlation between dry strength and density is rather poor for this particular fibre type (Figure 9b), suggesting that the inelastic behaviour after yielding of the fibre network is important for dry strength. All in all, it seems that the mechanisms underlying dry strength are much more complex than the inter-diffusion mechanism previously discussed in the case of wet strength. For example, in Figure 9b, there is a much higher dry strength value for a particular pressing condition corresponding to 62% solids content. In this case, the parallel measurements have very good reproducibility. Curiously, the elastic modulus, calculated from the same measurement curves, does not differ from the general trend observed for other conditions, as shown in Figure 9a. It is possible that the dry strength (and associated inelastic straining) is more sensitive to overheating of the fibre polymers than the wet strength. Thus, maximising dry strength may require a delicate balance of temperature and process dynamics for optimal moisture removal during hot-pressing. Generally, inelastic deformations are controlled by amorphous fibre components such as hemicelluloses, whose mechanical behaviour changes dramatically with varying moisture content and temperature [27,28,29].

**Figure 10:**
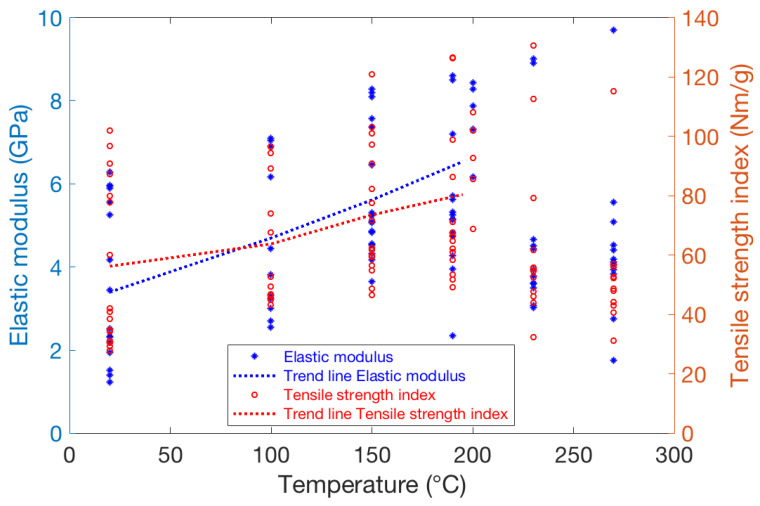
Elastic modulus and dry tensile strength index for a wide data set of sheets with uniform fibre orientation and different furnishes. One should notice that samples above 200 °C have been pressed using the steel-belt press, and those in the range of 20−200 °C have been pressed with cylinder press. The dashed trend lines describe the average behaviour for varied pressing temperatures.

The deterioration temperatures of cellulose and hemicellulose differ slightly, and some differences for the high-temperature behaviour of elastic modulus and dry tensile strength could be expected. The hemicelluloses degrade at 230–315 °C, whereas lignin decomposes over a broader temperature range of 200–500 °C [30,31]. However, as seen in Figure 10 for varied pulp types, both elastic modulus and dry strength peak around 150–200 °C, followed up by a decrease for most cases when further increasing the temperature. In other words, the above slight differences in polymer degradation do not seem to change the big picture concerning the mechanical behaviour of materials with different pulp types. The only exceptions are a few kraft pulp samples, which contain some lignin that might shield hemicelluloses, and for which a similar decrease of mechanical properties beyond 200 °C is not observed.

Standard carbohydrate analysis (performed with SCAN-CM 71:09) of some of the samples pressed at 20 °C and 270 °C showed a small reduction in hemicellulose content (calculated according to KA 10.314) caused by hot-pressing. The samples containing nearly 100% carbohydrate showed the largest decrease in hemicelluloses (less than 10% decrease), perhaps due to the lack of “protecting” lignin. However, in general, the changes are rather small in all cases, which is in agreement with earlier findings in wood welding studies [32]. This means that it is not possible to explain the decrease in mechanical properties caused by hot-pressing, as seen in Figure 10, solely by observing the changes in hemicellulose content.

## 4. Discussion

Hot-pressing often produces a significant increase in the wet tensile strength of paper webs. This effect is strongest when the lignin content of the fibres exceeds 7–12% and the pressing temperature is as high as possible. It is possible that there is an optimum range of lignin contents for wet strength. The relative change in wet strength with temperature is similar for very different pulps, which is explained by a similar lignin inter-diffusion mechanism that strengthens inter-fibre bonding under wet conditions. The strong exponential temperature dependence of the diffusion rate appears to overrule other factors such as pressing time or changes in network density (affected by nip pressure) in determining wet strength levels. This suggests that the wet-strengthening mechanism described above is not sensitive to the amount of lignin diffusing into the bond region between fibres. Even a very thin layer of lignin is sufficient to glue fibre surfaces together so that the bond formed is water resistant. However, it appears that a lignin content of at least 7% is required to cover the surfaces well enough for the wet strength improvement to reach its full potential.

When investigating wet strength for TMP papers with varying initial solids content, the best results were obtained when pressing an initially dry web, which is also expected to have the highest web temperature. It should be noted that the theoretical intra-fibre vapour pressure can become very high, several tens of bars, when the temperature is 200 °C or higher and the solids content is below 80% (see Appendix A). This high internal pressure in the fibre walls does not appear to accelerate lignin transfer, at least when considering the observed changes in wet strength at different solids contents. In other words, lignin and water transport mechanisms seem to decouple from one another. This is very interesting since it is known that the presence of water critically affects the softening (i.e., Tg) of lignin.

The dry tensile strength of hot-pressed TMP paper shows a very complex behaviour under varying process conditions. Rather than having clear trends with varying solids content or temperature, certain conditions appear to be more optimal than others in unexpected ways. This behaviour differs depending on the pulp being pressed, e.g., mechanical pulp or chemical pulp, so it is difficult to draw general conclusions. However, it seems that softening, e.g., by water or sulfonation [13], is important for dry strength. It should be noted that dry strength is quite high even without pressing, so the relative changes are smaller than for wet strength. In addition, polymer degradation can degrade strength at high temperatures. Therefore, evaporation of water in the fibre walls can appropriately control the temperature rise and prevent polymer degradation. Perhaps the best conditions consist of pressing times and temperatures that are just sufficient to evaporate most of the water from the fibres but do not cause over-drying or heating of the fibres that degrades their strength properties.

## 5. Conclusions

The main findings in this study are highlighted below:

- Hot-pressing does not cause a noticeable change in density in the fibre walls, despite a large reduction in network porosity and mean pore size.
- The wet strength increases with increasing pressing temperature. The stronger bonding of the wet fibres can be explained by inter-diffusion of lignin macromolecules (with an activation energy around 26 kJ mol^−1^) after lignin softening. The associated exponential acceleration of diffusion with temperature dominates the setting of wet strength over other factors such as process dynamics or final material density.
- The highest solids content before hot-pressing for the TMP sheets was found to give the highest values for wet strength. A possible explanation for this is that when a drier sheet is pressed, a higher temperature is reached, which accelerates the inter-diffusion of the lignin and thus enhance bonding. No significant correlation was observed between the varied solids content before pressing and dry strength.
- The elastic modulus increases with the increasing density of the sheets after hot-pressing, as expected. On the other hand, the dry strength does not show the same trend, indicating that the inelastic behaviour after yielding is responsible for the observed differences among the trial points.
- For dry strength and elastic modulus, the optimum pressing temperature is lower than for wet strength due to the degradation of hemicelluloses.

## Figures and Tables

**Table 1:** Effective density of the TMP sheets obtained from the X-ray microtomography (CT) compared with the standard measurement.

Sample	Effective Sheet Density (CT)	Sheet Density (ISO 534)
Unpressed	367 kg/m^3^	313 kg/m^3^
Pressed 190 °C	955 kg/m^3^	694 kg/m^3^
Pressed 270 °C	1000 kg/m^3^	734 kg/m^3^

**Figure A1:**
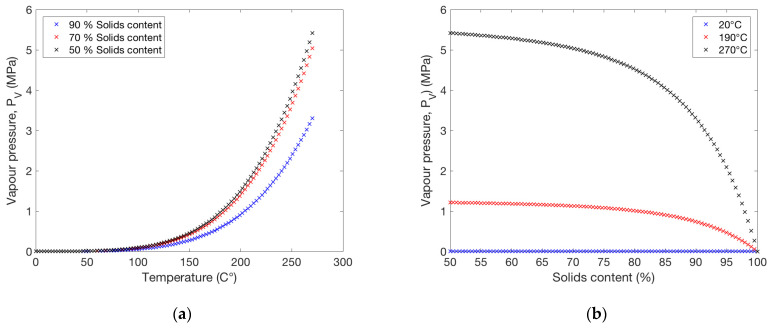
(**a**) Vapour pressure as a function of temperature for three different levels of solids content, 90%, 70%, and 50%. (**b**) Vapour pressure as a function of solids content for three different temperature levels, 20 °C, 190 °C, and 270 °C.

## Data Availability

The data in this study are available on request from the corresponding author.

## Appendix A

The vapour pressure $P_{v}$ inside a fibre wall at elevated temperatures can be estimated based on the theory developed by Flory and Huggins [33]. We assume that all water is bound to the (hemi)cellulose gel. In a binary polymeric solution (w water, c cellulose) [33], the vapour pressure $P_{v}$ is given by

(A1) $$ln\left(\frac{P_{v}}{P_{0}}\right)=\ln\left(\phi_{w}\right)+\left(1-\frac{v_{w}}{v_{c}}\right)\phi_{c}+\chi \phi_{c}^{2}$$

where $P_{0}$ is the vapour pressure of pure solvent, approximated by the Antoine equation

(A2) $${log}_{10}P_{0}=A-\frac{B}{C+T};\;A=8.071,\;B=1731\;{}^{\circ}C,\;C=233.4\;{}^{\circ}C\;(P_{0}\;in\;Torr)$$

In Equation (A1), $\phi_{i}$ is volume fraction, $v_{i}$ is molar volume (molar mass divided by mass density), and χ is Flory–Huggins interaction parameter (for cellulose at moderate water contents). According to reference [34], the specific volume (or density) of cellulose does not vary much with temperature up to 190 °C if the pressure remains below 20 MPa. Assuming that the lignin content of fibre is $c_{l}$, the cellulose content is $1-c_{l}$. The volumes of the different components are (assuming bulk phase dominates the volume for both components when the moisture content is high).

(A3) $$V_{i}=\frac{M_{i}}{\rho_{i}}$$

For cellulose, we obtain the volume

(A4) $$V_{c}=\frac{\left(1-c_{l}\right)M_{f}}{\rho_{c}}$$

where $M_{f}$ is dry fibre mass and $\rho_{c}$ is cellulose density. The water mass in terms of moisture content mc and dry fibre mass $M_{f}$ becomes

(A5) $$M_{w}=\frac{mc\;M_{f}}{100-mc}$$

leading to the water volume

(A6) $$V_{w}=\frac{mc\;M_{f}}{\left(100-mc\right)\rho_{W}}$$

where $\rho_{w}$ is water density. The total volume of the water-cellulose gel can be approximated as

(A7) $$V_{T}\approx V_{c}+V_{w}=\left[\frac{1-c_{l}}{\rho_{c}}+\frac{mc}{\left(100-mc\right)\rho_{w}}\right]M_{f}=\frac{\left(1-c_{l}\right)\left(100-mc\right)\rho_{w}+mc\rho_{c}}{\rho_{c}\left(100-mc\right)\rho_{w}}M_{f}$$

Thus, we can write volume fractions as

(A8) $$\phi_{w}=\frac{V_{w}}{V_{T}}\approx \frac{mc\;\rho_{c}}{\left(1-c_{l}\right)\left(100-mc\right)\rho_{w}+mc\rho_{c}}$$

(A9) $$\phi_{c}=\frac{V_{c}}{V_{T}}\approx \frac{\left(1-c_{l}\right)\left(100-mc\right)\;\rho_{w}}{\left(1-c_{l}\right)\left(100-mc\right)\rho_{w}+mc\rho_{c}}$$

In Figure A1 we show the behaviour of vapour pressure for varied temperatures and solids contents. The used values of the parameters are presented in Table A1.

**Table A1:** The values of the parameters used for plotting the vapour pressure as a function of temperature and solids content in Figure A1.

Parameter	Value
Molar volume of water ($v_{w}$)	18.02 cm^3^/mol
Molar volume of cellulose ($v_{c}$)	101.3 cm^3^/mol
Flory–Huggins interaction parameter (χ)	0.67 [35]
Density of water ($\rho_{w}$)	1000 kg/m^3^
Density of (crystal) cellulose ($\rho_{c}$)	1600 kg/m^3^
Lignin content of fibre ($c_{l}$)	25%
